# Supplementary material for: Survey report on keratoplasty in China: A 5-year review from 2014 to 2018
Source: PLoS One. 2020 Oct 15;15(10):e0239939. doi: 10.1371/journal.pone.0239939 (PMC7561196; doi:10.1371/journal.pone.0239939)
Supplement: S1 Table — (PDF) [file pone.0239939.s001.pdf]

全国角膜移植手术调查登记表 （中文）

单位名称： **填报人员：** **联系电话：**

序号	时间	住院号	姓名	性别	接受手术年龄（岁/月）	术前诊断	手术方式	供体保存方式	并发症	其他
1	2014									
2	2015									
3	2016									
4	2017									
5	2018									
……										

填表说明	1. 时间：按年限分开统计，表格中时间用阿拉伯数字，数字后不写单位。例：2017 或 2018；
	2. 姓名：为了保护患者隐私，可简写或用字母表示；
	3. 手术年龄：用阿拉伯数字，整岁数字后面不写单位，不满一岁按实际月龄填报
	4. 手术方式：请按以下方式填写：PK, ALK, EK, 干细胞移植、三联
	5. 供体保存方式：请按以下方式填写：中期保存、甘油脱水保存、湿房保存。其他情况请备注。
备注	本统计表请于 3 月 25 号前以邮件形式反馈至指定邮箱（sdykzkzx@163.com），谢谢配合。

Questionnaire of Keratoplasty in China (English)

Hospital name: **Completed by:** **Phone number:**

No.	Year	Hospitalization No.	Name	Gender	Patient's Age （year/month）	Disease	Surgical Technique	Cornea Preservation Method	Complication	Others
1	2014									
2	2015									
3	2016									
4	2017									
5	2018									
……										

Explanation	1. Year: Please provide the information of keratoplasty in 2014, 2015, 2016, 2017 and 2018 separately.
	2. Name: To protect the privacy of the patients, abbreviations or letters instead of their real name can be used.
	3. Patient’s Age: Please use Arabic numerals. For patients under one year old, please use unit “month” instead of “year”.
	4. Surgical Technique: Penetrating keratoplasty, anterior lamellar keratoplasty, endothelial keratoplasty, keratoprosthesis, keratolimbal allograft.
	5. Cornea Preservation Method: Mid-term storage, dehydration, moist chamber. Please write down other conditions in “Others”.
Note	Please send the questionnaire back to sdykzkzx@163.com before March 25. Thank you for your cooperation.
