# Supplementary material for: Survey report on keratoplasty in China: A 5-year review from 2014 to 2018
Source: PLoS One. 2020 Oct 15;15(10):e0239939. doi: 10.1371/journal.pone.0239939 (PMC7561196; doi:10.1371/journal.pone.0239939)
Supplement: Attachment — Submitted filename: Response to Reviewers.docx [file pone.0239939.s002.docx]

Dear editors and reviewers,

We are grateful to you for your time and constructive comments on our manuscript entitled "Survey report on keratoplasty in China: a 5-year review from 2014 to 2018". The comments and suggestions are very valuable and useful for improving the manuscript. We have carefully implemented the comments and suggestions and wish to submit this revised version for further consideration in the journal. Below, we also provide point-by-point responses explaining how we have addressed each of the comments.

Looking forward to hearing from you soon.

Thank you and with kindest regards.

Yours Sincerely,

Weiyun Shi

On behalf of co-authors

Journal Requirements:

When submitting your revision, we need you to address these additional requirements.

1. Please ensure that your manuscript meets PLOS ONE's style requirements, including those for file naming. The PLOS ONE style templates can be found at https://journals.plos.org/plosone/s/file?id=wjVg/PLOSOne_formatting_sample_main_body.pdf and <https://journals.plos.org/plosone/s/file?id=ba62/PLOSOne_formatting_sample_title_authors_affiliations.pdf>

**Answer：** We have modified the manuscript according to the templates.

2. Please include additional information regarding the survey or questionnaire used in the study and ensure that you have provided sufficient details that others could replicate the analyses. For instance, if you developed a questionnaire as part of this study and it is not under a copyright more restrictive than CC-BY, please include a copy, in both the original language and English, as Supporting Information.

**Answer：** We have included a copy of the questionnaire in both Chinese and English as supporting information.

3. Please modify the title to ensure that it is meeting PLOS’ guidelines (https://journals.plos.org/plosone/s/submission-guidelines#loc-title). In particular, the title should be "specific, descriptive, concise, and comprehensible to readers outside the field" and in this case it is not informative and specific about your study's scope and methodology.

**Answer:** We have modified the title to “Survey report on keratoplasty in China: a 5-year review from 2014 to 2018” according to the guidelines. If the title should be further changed, would you please give us some suggestion? Thank you very much.

4. PLOS requires an ORCID iD for the corresponding author in Editorial Manager on papers submitted after December 6th, 2016. Please ensure that you have an ORCID iD and that it is validated in Editorial Manager. To do this, go to ‘Update my Information’ (in the upper left-hand corner of the main menu), and click on the Fetch/Validate link next to the ORCID field. This will take you to the ORCID site and allow you to create a new iD or authenticate a pre-existing iD in Editorial Manager. Please see the following video for instructions on linking an ORCID iD to your Editorial Manager account: <https://www.youtube.com/watch?v=_xcclfuvtxQ>

**Answer:** The ORCID ID of the corresponding author has been added. Thank you.

5. Please amend the manuscript submission data (via Edit Submission) to include authors:

Ting Huang2,3, Zhiqiang Pan2,4, Jie Wu2,5, Jianjiang Xu2,6, Jing Hong2,7,
Wei Chen,2,8, Huping Wu9, Qian Kang10, Lei Zhu2,11, Lingling Fu12, Liqiang Wang2,13,
Guigang Li2,14, Zhihong Deng15, Hong Zhang2,16, Hui Xu17, Qingliang Zhao18,
Hongshan Liu19, Linnong Wang2,20, Baihua Chen2,21, Xiuming Jin2,22, Minghai
Huang23, Jizhong Yang24, Minghong Gao2,25, Wentian Zhou26, Hanping Xie2,27, Yao
Fu2,28, Feng Wen29, Changbo Fu30, Shaozhen Zhao2,31, Yanning Yang2,32, Yanjiang
Fu33, Tao Yao34, Chaoqing Wang35, Xiaonan Sun36, Xiaowei Gao2,37, Maimaitiming
Reziwan38, Yingping Deng2,39, Jian Li40, Limei Liu 41, Bo Zeng42, Lianyun Bao43, Hua
Wang 2,44, Lijun Zhang2,45, Zhiyuan Li46, Zhijian Yin47, Yuechun Wen 48, Xiao Zheng
49, Liqun Du 50, Zhenping Huang 51, Xunlun Sheng 52, Hui Zhang2,53, Lizhong Chen 54,
Xiaoming Yan 2,55, Xiaowei Liu 56, Wenhui Liu 57, Yuan Liu 58, Liang Liang 59,
Pengcheng Wu 60, Lijun Qu61, Jinkui Cheng62, Hua Zhang63, Qige Qi64, Yangkyi
Tseten65, Jianping Ji3, Jin Yuan2, 3, Ying Jie4, Jun Xiang6, Yifei Huang2,13, Yuli
Yang2,27, Ying Li2,56, Yiyi Hou1, Tong Liu1, Lixin Xie.

**Answer:** Thank you very much. Information of all the authors has been added to the submission system.

6. Please include captions for your Supporting Information files at the end of your manuscript, and update any in-text citations to match accordingly. Please see our Supporting Information guidelines for more information: [http://journals.plos.org/plosone/s/supporting-information](about:blank).

**Answer:** Thank you very much. We have included the caption for the supporting information at the end of the manuscript and updated the in-text citation.


Reviewers' comments:

**Comments to the Author**

Reviewer #1: This is a very extensive study with valuable information. The paper is well written. I have the following comments/suggestions to improve the scientific content.
The information of donor cornea preservation is incomplete. The authors should elaborate this aspect by providing specific information about the type of corneal storage medium and describe what is meant by the dry method.

**Answer:** Thank you very much for raising this point. Limited by the requirements on article length, we have not given comprehensive information of donor cornea preservation in the manuscript that we submitted. In fact, there is much information that should be reported, so after careful discussion, in this revised manuscript, we decided to delete all the information about donor cornea preservation and in the near future, we will write an article reporting the survey results on cornea preservation and compare them with those in America.

The survey was based on a questionnaire. More details of the methodology of obtaining the information and the questions asked and collection of the responses can be provided.

**Answer:** Thank you very much for your suggestion. In the revised manuscript, we have added the questionnaire as supporting information.

Reviewer #2: Thank you for the opportunity to review this manuscript. It has the potential to be an important paper, as this is the first time such a comprehensive survey of keratoplasty in China has been attempted.

The current paper is a good draft, but I think there are multiple clarifications that need to be made before it can be published.

Background
1) many readers outside of China will not be familiar with the geography and the classification of provinces in China, so may I suggest that you explain
- how provinces and cities in China are categorised into "tiers"
- if possible, a map to show where the various participating hospitals are located - you may not be able to place all 64 hospitals on the map, but perhaps you can list the hospitals by geographical location?

**Answer:** Thank you very much for your suggestion. We agree with you on your opinion that many foreigners are not familiar with the classification of provinces in China, so in the manuscript, to avoid confusing the readers, we use “provincial-level administrative units” instead of terms like “province” and “municipality directly under the central government”.

Methods

2) Some detail needs to be provided on how corneas in China are obtained.
- I understand that use of foreign or imported tissue is highly restricted?
- the use of bio-engineered tissue is very interested, could you elaborate on how these tissues are engineered?
- proportion of fresh, versus frozen, versus bio-engineered tissue would give readers some context

**Answer:** Thank you very much for raising this point. As you said, the use of foreign and imported tissue is highly restricted. In China, the procurement, management and use of cornea tissue are more standardized in recent years. However, there is still a huge shortage of corneas in China, so ophthalmologists are making efforts to look for alternatives. Prof. Weiyun Shi and his team have made breakthrough in the field of bio-engineered cornea and published articles about decellularized porcine cornea (Shi, W, Zhou, Q, Gao, H, Li, S, Dong, M, & Wang, T, et al. (2019). Protectively decellularized porcine cornea versus human donor cornea for lamellar transplantation. *Advanced Functional Materials*, 29(37), 1902491.1-1902491.12. Dong, Muchen & Zhao, Long & Wang, Fuyan & Hu, Xiaoli & Li, Hua & Liu, Ting & Zhou, Qingjun & Shi, Weiyun. (2019). Rapid porcine corneal decellularization through the use of sodium N-lauroyl glutamate and supernuclease. Journal of Tissue Engineering. 10. 204173141987587. 10.1177/2041731419875876.). In our study, the main preservation methods of donor corneas were mid-term preservation (29 252, 79.89%), preservation by dehydration (5 864, 16.01%) and preservation in moist chamber (1 500, 4.10%). We found that there is much information that can be reported based on the results of the survey, so we decided to delete all the information about cornea procurement and preservation in this article and write an article thoroughly reporting the information.

3) a copy of the questionnaire used will be important, and included as an annex. An English translation would be appropriate for most readers.

**Answer:** Thank you very much for your suggestion. We have added a copy of questionnaire as supporting information.

4) Was there also information collected on graft survival, at least in the first year?

**Answer:** Thank you very much for raising this point. To be honest, we have not collected information on graft survival. In the future, when we do this survey, we will improve the questionnaire and collect information on CT.

5) Information on the number of surgeries done - lines 276 to 296 - is best summarised in a table rather than written in the text. Similarly with the gender and age distribution.

**Answer:** Thank you very much for your suggestion. We have expressed the information in a table.

6) Indications for surgery
- unfortunately, cornea leukoma is a rather rather term, unless there is a specific-definition used by the Chinese Cornea Society? Usually there is an underlying cause - congenital, trauma, infection. Would the authors be able to give further details?

**Answer:** Thank you very much for raising this point. According to the survey results collected from the hospitals, we can not give detailed information on the underlying causes. We have called the hospital and they said that for some patients, the underlying causes were hard to diagnosed, so cornea leukoma was the indication for their surgeries. The cases whose causes of cornea leukoma were diagnosed have been included in the corresponding indications.

- similarly, cornea perforation is very common, but it would be better to further tell readers if this is due to trauma or infection, ot other causes

**Answer:** Thank you very much for raising this point. According to the survey results collected from the hospitals, we can not give detailed information on the underlying causes. We have called the hospital and they said that for some patients, the underlying causes were hard to diagnosed, so cornea perforation was the indication for their surgeries. The cases whose causes of cornea perforation were diagnosed have been included in the corresponding indications.

- I am surprised bacteria keratitis is such a common indication, but fungal keratitis is not, even though fungal infections are as high as bacterial keratitis in China. Is this practice pattern (meaning that surgery is not done for fungal keratitis), or is it because it is classified under other indications - eg perforations?

**Answer:** Thank you very much for your question. In the survey, fungal keratitis was not classified under other indications.

7) ALK indications
- curious to see small numbers of corneal perforation - are these patch grafts?
- similarly I see ALK for pseuophakic bullous keratopathy

**Answer:** Thank you very much for raising this point. The main reason for ALK performed for patients with corneal perforation and pseuophakic bullous keratopathy was the shortage of fresh cornea tissue in China, so ALK was used as an alternative to save the patients’ eyes. Besides, for patients with pseuophakic bullous keratopathy, PK and EK are the preferred methods, but without qualified corneas, ALK can be performed to alleviate their pain.

8) Discussion
- Could the authors comment how the availability of tissue also affects practice patterns?
for example, there could be much more ALK done in China vs the USA because of the lack of healthy fresh tissue for PK and EK

**Answer:** Thank you very much for raising this point. The cornea types that are available do influence the surgery types the ophthalmologists selected. Benefiting from the questions and suggestions from the reviewers, we realized that we need to reporting more valuable information on cornea procurement and preservation, which influences the surgery patterns selected. We should not waste the hard-won information from the survey, so in addition to this article, we decided to write another article reporting this aspect comprehensively.

- could authors also comment on what more can be done to improve the CT situation - what this paper shows is that much more CT needs to be done to have any hope of helping the 3 million in China with cornea blindness - is bioengineered cornea a good option? what about training and facilities - 64 hospitals perform transplants, but many are done in smaller units and do much fewer transplants than the top 5 hospitals

**Answer:** Thank you very much for raising these points. Measures should be taken to improve the CT situation in China. For example, the society can pay more attention to the publicity of cornea donation and the government and professional organizations can carry out more training. A course on corneal transplantation led by Prof. Weiyun Shi has training more than 200 ophthalmologists.

Bio-engineered cornea is a good option, which can ease the situation of cornea shortage in China. Bio-engineered corneas do not need to be stored in eye banks, so it is more convenient to use.

To narrow the gap between the hospitals, we think that developing economy is of great importance.

Reviewer #3: This paper aims to analyze the trend of corneal transplants in China, focusing in particular on the diffusion of lamellar techniques and surgical indications and to compare the results with the trend in United States (US).
I found the manuscript interesting and original, since few data are available about corneal transplant in China.
I suggests the authors to include the survey as supplemental material.
Moreover, I suggest to include in the discussion the results of similar studies conducted in other countries, in addiction to the US situation.
The sentence at page 21 ,lines 377-328, should be corrected, since only EK surpassed the number of PK in US since 2011 (ALK is still inferior to PK).

**Answer:** Thank you very much for your careful review. We have added the questionnaire as supporting information.

Comparing the results in China with the results in other countries can better reflect the differences and provide more information for readers, but this survey was designed referring to the EBAA report, so we only compared the results with those in America. At the beginning of the study, we have compared reports and articles of different countries in this field. EBAA was the one who reported comprehensively and annually, so we decided to compare the situation in China with the situation in America.

In the sentence at page 21, lines 377-378, “various LK” means ALK and EK. Thank you for pointing out. To express the meaning more clearly, we have modified the expression in the sentence.
