# Supplementary material for: Survey report on keratoplasty in China: A 5-year review from 2014 to 2018
Source: PLoS One. 2020 Oct 15;15(10):e0239939. doi: 10.1371/journal.pone.0239939 (PMC7561196; doi:10.1371/journal.pone.0239939)
Supplement: Attachment — Submitted filename: Response to Reviewers.docx [file pone.0239939.s003.docx]

Dear Dr. Liu and reviewers,

We would like to give our great thanks to you and all the reviewer, for your valuable comments on our manuscript entitled "Survey report on keratoplasty in China: a 5-year review from 2014 to 2018". Thank you for your suggestions and efforts to make the article better. We have carefully implemented the comments and suggestions and wish to submit this revised version for further consideration in the journal. Below, we provide point-by-point responses explaining how we have addressed each of the comments.

Looking forward to hearing from you soon.

Thank you and with kindest regards.

Yours Sincerely,

Weiyun Shi

On behalf of co-authors

1. Reviewer #1: The authors have addressed the concerns raised. Minor editorial corrections and language changes are required. For example, one-center can be changed to single center. Line 468-471 the sentence "Overall, in China, the geographical ...weak in CT" in the conclusion needs to be re-written as it is not clear.

**Answer:** Thank you very much for your time and suggestions. We have read the article carefully and thoroughly to avoid typographical and grammatical errors. “One-center” has been changed to “single center”. The sentence has been rewritten.

2. Reviewer #2: Thank you once again for the opportunity to review this paper, as I was Reviewer 2 for the first submission. I appreciate that the authors have made some of the amendments as I have suggested, including summarizing the data in tables. Other information that I felt would have made this a more complete paper appears to not be available, based on the methods and on how the questionnaire was designed. I think it is therefore important to include a section of the paper to address the limitations of the study - namely that this was a retrospective study where information is incomplete, diagnoses and classification of indications are not standardized (eg the meaning of cornea leukoma), and that outcome data in terms of graft survival was not obtained.

**Answer:** Thank you very much for your valuable suggestions on the previous manuscripts. We benefit a lot from your comments and we have modified the article accordingly. No doubt there are limitations in the questionnaire and they are hard to be corrected for the survey from 2014 to 2018. Therefore, we adopted your advice and added sentences at the end of the manuscript to address the limitations of the study. We appreciate your efforts to make the article and the questionnaire better.
